# Comparative Effects of Alpha- and Gamma-Tocopherol on Mitochondrial Functions in Alzheimer’s Disease In Vitro Model

**DOI:** 10.1038/s41598-020-65570-4

**Published:** 2020-06-02

**Authors:** Aslina Pahrudin Arrozi, Siti Nur Syazwani Shukri, Wan Zurinah Wan Ngah, Yasmin Anum Mohd Yusof, Mohd Hanafi Ahmad Damanhuri, Faizul Jaafar, Suzana Makpol

**Affiliations:** Department of Biochemistry, Level 17, Preclinical Building, Universiti Kebangsaan Malaysia Medical Centre, Jalan Yaacob Latif, Bandar Tun Razak, Cheras 56000 Kuala Lumpur, Malaysia

**Keywords:** Preclinical research, Translational research

## Abstract

Vitamin E acts as an antioxidant and reduces the level of reactive oxygen species (ROS) in Alzheimer’s disease (AD). Alpha-tocopherol (ATF) is the most widely studied form of vitamin E besides gamma-tocopherol (GTF) which also shows beneficial effects in AD. The levels of amyloid-beta (Aβ) and amyloid precursor protein (APP) increased in the brains of AD patients, and mutations in the *APP* gene are known to enhance the production of Aβ. Mitochondrial function was shown to be affected by the increased level of Aβ and may induce cell death. Here, we aimed to compare the effects of ATF and GTF on their ability to reduce Aβ level, modulate mitochondrial function and reduce the apoptosis marker in SH-SY5Y cells stably transfected with the wild-type or mutant form of the *APP* gene. The Aβ level was measured by ELISA, the mitochondrial ROS and ATP level were quantified by fluorescence and luciferase assay respectively whereas the complex V enzyme activity was measured by spectrophotometry. The expressions of genes involved in the regulation of mitochondrial membrane permeability such as voltage dependent anion channel *(VDAC1)*, adenine nucleotide translocase *(ANT)*, and cyclophilin D *(CYPD)* were determined by quantitative real-time polymerase chain reaction (qRT-PCR), while the expressions of cyclophilin D (CypD), cytochrome c, Bcl2 associated X (BAX), B cell lymphoma-2 (Bcl-2), and pro-caspase-3 were determined by western blot. Our results showed that mitochondrial ROS level was elevated accompanied by decreased ATP level and complex V enzyme activity in SH-SY5Y cells expressing the mutant *APP* gene (p < 0.05). Treatment with both ATF and GTF reduced the mitochondrial ROS level with maximum reduction was observed in the cells treated with high concentrations of ATF and GTF (p < 0.05). However, only GTF at 80 µM significantly increase the ATP level and complex V enzyme activity (p < 0.05). *VDAC1* and *CYPD* were downregulated and CypD protein was significantly overexpressed in cells transfected with the wild-type (WT) and mutant *APP* gene (p < 0.05). Cytochrome c release, the ratio of BAX/Bcl-2, and pro-caspase-3 expression increased in cells expressing mutated *APP* gene (p < 0.05). The expression of CypD and pro-caspase 3 protein, and the ratio of BAX/Bcl-2 were increased in the following order; SH-SY5Y-APP-WT < SH-SY5Y-APP Swe <SH-SY5Y-APP Swe/Ind. Treatment with both ATF and GTF reduced the release of cytochrome c and the ratio of BAX/Bcl-2. However, only GTF significantly reduced the expression of CypD and pro-caspase-3, suggestive of its unique role in AD. In conclusion, GTF has an effect that was not shown by ATF and thus suggest its potential role in the development of therapeutic agents for AD.

## Introduction

Among the eight soluble isoforms of vitamin E (α-, β-, γ-, and δ-tocopherol and α-, β-, γ-, and δ-tocotrienol), our body efficiently absorbs α-tocopherol (ATF) owing to its high affinity for alpha-tocopherol transfer protein (α-TTP), a vitamin E transporter protein^1^. ATF has received the most attention over the past decades and is the major isoform of vitamin E known for its protective functions against diseases. In Alzheimer’s disease (AD), ATF acts in response to the increase in oxidative stress and serves as a potent antioxidant by scavenging and eliminating free radicals such as reactive oxygen species (ROS) and hydrogen peroxide^2^. However, the biological activities of ATF are not limited to its antioxidant actions as ATF is also known to influence cell signaling and expressions of genes involved in the development of AD^3,4^.

Several studies have demonstrated the unique functions and benefits of γ-tocopherol (GTF) that distinguish it from alpha tocopherol, regardless of its low bioavailability in the body. GTF reduces the oxidative stress in the brain induced by reactive nitrogen species (RNS) more efficiently than ATF^5^. Several genes involved in the clearance of amyloid-beta (Aβ) protein were strongly affected in the hippocampus of male albino rats fed with dietary vitamin E that mainly comprises GTF, suggestive of the protective effect of GTF on AD progression^6^. Other studies have shown an inverse association between AD and subjects that consumed high proportion of vitamin E from food (a mixture of tocopherols) but not those that consumed ATF alone. Furthermore, ATF and GTF have shown inverse association with both AD and cognitive decline that were similar in magnitude^7^. These evidences showed that the distinguished features of GTF may be beneficial to human health in ways not recognized previously and more research need to be carried out in order to highlight the potential of GTF as compared to ATF. Furthermore, the previous study only showed the capability of ATF and GTF as an antioxidant and signaling molecule however, their potential in modulating specific organelle function such as mitochondria remains to be elucidated.

Accumulation of Aβ protein in the brain of patients with AD is one of the pathological hallmarks of the disease^8^. Mutations in amyloid precursor protein (APP), such as Swedish (Swe) and Indiana (Ind) mutations, result in increased production of Aβ and the subsequent rise in the level of Aβ^9,10^. Our previous findings on the SH-SY5Y cells stably transfected with the wild-type (WT) or mutant *APP* gene showed an increase in the ratio of Aβ42/40 in the following order; SH-SY5Y-APP WT < SH-SY5Y-APP Swe <SH-SY5Y-APP Swe/Ind^11^. In a previous study, high Aβ levels has been shown to promote mitochondrion-induced production of ROS and dysfunction of the mitochondria itself further triggers the formation of Aβ^12^. The ability of Aβ to interact with many proteins when present at high concentrations may cause abnormal changes in the cells. Increasing evidence indicates mitochondria as an important target of Aβ as Aβ can accumulate and bind to proteins in the mitochondria^13^.

A study by Du *et al*.^14^ showed that deficiency of cylophilin D (CypD) protein, a key protein for regulating the mitochondrial membrane permeability, protects against Aβ-mediated mitochondrial dysfunction suggesting a possible direct interaction between Aβ and CypD. Besides CypD, voltage-dependent anion channel (VDAC1) and adenine nucleotide translocator (ANT) also regulates mitochondrial membrane permeability. However, a study using VDAC1 knockout mice and mice with ANT deficiency showed that these proteins did not affect mitochondrial membrane permeability thus clarifying its role in the formation of anion channel but not limiting the activity of the channel^15,16^.

The increase in the mitochondrial membrane permeability due to mitochondrial swelling may trigger the release of cytochrome c that later activates Bax, Bcl-2 and caspase-3 leading to apoptosis^17^. Clementi *et al*.^18^ reported that human neuroblastoma cell line treated with Aβ42 undergoes apoptosis via the increase in the ratio of BAX/Bcl-2^19^. Besides, the fate of cell death is also determined by the level of intracellular ATP. In a previous study, Aβ was shown to bind to subunit α of F_0_ F_1_ of complex V enzyme and cause inhibition of the enzyme activity that eventually led to a reduction in ATP production^20^. Based on these findings, the occurrence of cell death may be triggered by mitochondrial dysfunction or abnormalities induced by Aβ. Therefore, in this study, we aimed to compare the potential effects of ATF and GTF in decreasing the level of Aβ and their modulation on mitochondrial function by determining mitochondrial ROS and ATP production, complex V enzyme activity and mitochondrial membrane permeability as well as apoptosis markers in SH-SY5Y cells stably transfected with the wild-type or mutant form of *APP* gene. This study may reveal the potential application of both vitamin E isoforms in the development of therapeutic strategies in Alzheimer’s disease by targeting the mitochondria organelle.

## Methods

### SH-SY5Y cells stably expressing the WT or mutant *APP* Gene

The development of SH-SY5Y cells stably expressing the WT or mutant *APP* gene has been described in our previous study^11^. This study used the same batch of cells as described previously in which the ratio of Aβ42/40 was increased in the following order SH-SY5Y-APP WT < SH-SY5Y-APP Swe < SH-SY5Y-APP Swe/Ind. Briefly, SH-SY5Y cells were transfected with three different plasmids carrying the wild-type (WT), Swedish (Swe), or Swedish/Indiana (Swe/Ind) form of *APP* gene using Lipofectamine 3000. After 72 h of transfection, the positive green fluorescent protein (GFP)-expressing cells were selected using a selection medium containing 400 µg/mL of geneticin (G418). The steady state of APP gene and protein expression as well as the production of secreted Aβ42/Aβ40 were measured for verification.

### Cell culture

Non transfected SH-SY5Y cells were cultured in a complete culture medium (CCM) containing 1:1 ratio of Dulbecco’s modified Eagle’s medium (DMEM) and Ham’s F-12 medium supplemented with 1% penicillin/streptomycin (Gibco, USA) and 10% fetal bovine serum (FBS) (HyClone, USA). Stably transfected SH-SY5Y cells were cultured in CCM without penicillin/streptomycin and with 400 µg/mL of geneticin (Gibco, USA). All cell types were cultured in a humidified atmosphere of 5% CO_2_ at 37 °C.

### Preparation of tocopherol isomers

The stock solutions of ATF (98.9% pure d-ATF) and GTF (95% pure d-GTF) (ChromaDex, USA) were freshly prepared in 100% ethanol to a final stock solution of 0.5 M and stored at −20 °C for not more than 1 month. Prior to treatment, 15 µl of each isomer from the stock solution was incubated overnight with 20 µl FBS at 37 °C. Then, the isomer stock solution was diluted to 0.1 M with 18 µl of CCM and 21 µl of 100% ethanol. The isomer stock solution was further diluted to 0.05 M with 72 µl of mixture of CCM and 100% ethanol in 1:1 ratio (50% ethanol). Subsequently, the isomer stock solution was diluted to 0.025 M with 146 µl of CCM only. Next, 60 µl of this stock solution was taken out and added into a new micro centrifuge tube. Then, 240 µl CCM was added into the tube to prepare 0.005 M stock solution. Several stocks solution were then prepared from the stock solution of 0.005 M. Finally, the desired working concentrations of each isomer (5, 80, and 100 µM) were prepared from their respective stock solution and this is the final concentration of each tocopherol isomer in the culture media added to the cells. The final concentration of ethanol and FBS were kept constant at 0.1% and 0.027% respectively (calculated from the overnight incubation with 20 µl FBS). Tocopherol treatment was carried out for 24 h.

### Cell viability assay

Cell viability was assessed with CellTiter 96 Aqueous Non-Radioactive Cell Proliferation Assay (Promega, USA). The kit consists of 3-(4,5-dimethylthiazol-2-yl)-5-(3-carboxymethoxyphenyl) 2-(4-sulphonyl)-2H-tetrazolium (MTS) and the electron coupling agent phenazine methosulphate (PMS). In active cells, the dehydrogenase enzyme reduced the MTS compound into a formazan product that is soluble in the medium and the amount of this coloured formazan product is proportional to the number of viable cells. Briefly, MTS solution was premixed with media at the ratio of 2:3 before adding 50 µL of the solution to each well of the cells and incubated in humidified incubator at 37 °C in 5% CO_2_ for 2–4 h. The quantity of formazan product was determined by measuring the absorbance at 490 nm with a microplate reader (Enspire, Perkin Elmer, USA).

### Aβ42 sandwich ELISA assay

Conditioned media was collected from the cells and protein inhibitors were added to the media to prevent degradation of Aβ protein. The concentration of Aβ42/40 was determined by using human Aβ42 or Aβ40 ELISA kit (Elabscience, China) according to the manufacturer’s instructions.

### Isolation of cytoplasmic proteins from mitochondrial proteins

The isolation of cytoplasmic proteins from mitochondrial proteins was carried out using ProteinExt Mammalian Mitochondrial Isolation kit (TransGen Biotech, China) according to manufacturer’s instructions. The purity of the cytoplasmic fraction was assessed using antibody Cox 4 (Santa Cruz, USA), a transmembrane marker of mitochondria.

### Fluorescence quantification

The quantification of mitochondrial ROS production was performed using Elite^TM^ mitochondrial ROS activity assay kit (eEnzyme LLC, Gaitherburg, MD, USA). The membrane-permeable non-fluorescent ROS sensor provided in the kit quickly penetrates into mitochondria and generates very strong fluorescence signals upon reaction with mitochondrial ROS (especially superoxide and hydroxyl radicals). Briefly, cells were incubated with 1× Elite^TM^ ROS deep-red solution for 30 min in a humidified atmosphere of 5% CO_2_ at 37 °C. The fluorescence intensity was measured using a microplate reader (Enspire, Perkin Elmer, USA) at excitation/emission wavelengths (Ex/Em) of 650/675 nm. Data was normalized to the protein content (µg) in each cells group.

### ATP level quantification

The ATP level was measured by using ATP Assay kit (Merck Millipore, USA). The kit contains Nuclear Releasing Reagent (NRR) and ATP Monitoring Enzyme (AME). The luciferase enzyme catalyzes the formation of light in the presence of ATP and luciferin. The intensity of the light was directly proportional to the ATP level production in the cells. Briefly, the cells were plated in 96-well plate. After 24 h treatment, the conditioned media was removed and 100 µL NRR was added. The cells were incubated for 5 min at room temperature with gentle shaking. Then, 1 µL AME was added to the cells and the plate was read using a microplate reader (Enspire, Perkin Elmer, USA) under luminescence. Data was normalized to the protein content (µg) in each cells group.

### Complex V enzyme activity assay

The enzyme activity of complex V was measured using Mitochondrial Complex V (ATP Synthase) Activity Assay kit (Merck Millipore, USA). The cells were plated in the 35 mm culture dish and after 24 h treatment, the ATP level was determined following the manufacturer’s protocol.

### Quantitative real-time polymerase chain reaction (qRT-PCR)

Total RNA was extracted using TRI reagent (Life Technologies, USA) and the expressions of *VDAC1, ANT* and *CYPD* were determined by quantitative reverse-transcription polymerase chain reaction (qRT-PCR) using one-step KAPA SYBR FAST qPCR kit (Kapa Biosystems, USA) according to manufacturer’s instructions. Total RNA and primer concentration were standardized to 100 ng and 400 nM, respectively. The primer sequences were as follows: Glyceraldehyde-3-phosphate dehydrogenase (*GAPDH*) forward 5′-TCCCTGAGCTGAACGGGAAG-3′ and reverse 5′-GGAGGAGTGGGTGTCGCTGT-3′; *VDAC1* forward 5′-GCCAAGTATCAGATTGACCC-3′ and reverse 5′-CTTGAAATTCCAGTCCTAGACC-3′; *ANT* forward 5′-AGTTCTGGCGCTACTTTGCT-3′ and reverse 5′-GCCTTGGACAGAGACGTTGA-3′; and *CYPD* forward 5′-CACCTTCCACAGGGTGATCC-3′ and reverse 5′-AGCAACAGTGTAGCGCAATG-3′. PCR reactions were carried out in a Bio-Rad iQ5 Cycler (Hercules, USA) with the following program: cDNA synthesis for 5 min at 42 °C; pre-denaturation for 4 min at 95 °C; and PCR amplification for 40 cycles of 3 s at 95 °C and 20 s at 60 °C. These reactions were followed by a melt curve analysis to determine the reaction specificity and expression of each target gene. The expression level of each gene was normalized to that of *GAPDH*. The relative expression value (REV) was calculated using the 2^−ΔΔCt^ method of relative quantification and the following equation:

$${\rm{REV}}={2}^{{\rm{Ct}}{\rm{value}}{\rm{of}}{\rm{GAPDH}}\mbox{--}{\rm{Ct}}{\rm{value}}{\rm{of}}{\rm{the}}{\rm{gene}}{\rm{of}}{\rm{interest}}}$$

### Western blot analysis

Cells were washed in ice-cold phosphate-buffered saline (PBS) and detached by scraping in a cell lysis buffer. Cell extracts were collected and stored at −80 °C. Protein concentration was measured with Bradford assay. Electrophoresis and blotting were performed using Mini-PROTEAN® Tetra Cell system (Bio-Rad, USA) according to manufacturer’s instructions. Denatured samples with an equal amount of protein per lane (20 μg) were separated on gradient gels (10% sodium dodecyl sulfate polyacrylamide gel electrophoresis [SDS-PAGE] gel except for cytochrome c detection, which was performed using 15% gel), electrotransferred on polyvinyl difluoride (PVDF) membranes (PALL, USA), and blocked in 5% skimmed milk for 1 h at room temperature. The membranes were overnight incubated with primary antibodies at 4 °C. The list of primary antibodies used is as follows: goat anti-cyclophilin-40, 1:1000 (AP16574PU-N) (BioCompare, USA), mouse anti-β-actin, 1:5000 (AC-15) (Santa Cruz, USA), mouse anti-cytochrome c, 1:1000 (EPM12250) (Elabscience, China), mouse anti-B-cell lymphoma-2 (Bcl-2), 1:1000, (100) (BioLegend, UK), mouse anti-caspase-3, 1:1000 (31A1067) (Santa Cruz, USA), and rabbit anti-BAX, 1:1000 (Poly6251) (BioLegend, UK). On the following day, membranes were washed several times in TPBS and incubated with a secondary antibody at 1:2000 dilution for 1 h at room temperature. The list of secondary antibodies is as follows: anti-rabbit horseradish peroxidase (HRP; 65–6120) (Thermo Fischer Scientific, USA), anti-mouse (ab136815) (Abcam, USA), and anti-goat (sc-2020) (Santa Cruz, USA). Peroxidase enzyme activity was determined using WesternBright™ Sirius Western blotting detection kit (Advansta, Menlo Park, CA, USA) and visualized on Gel-Doc Amersham Imager 600 (GE Healthcare Life Science, UK).

### Statistical analysis

Data obtained were expressed as mean ± standard deviation (SD) and statistical analysis was carried out using SPSS version 16. The analysis was performed using one-way analysis of variance (ANOVA) followed by Tukey’s post-hoc test for multiple comparisons. Values of p < 0.05 were considered significant.

### Consent for publication

All authors have read and approved the final manuscript. Authors give consent for publication in Scientific Reports.

## Results

### Treatment with ATF or GTF increased cell viability in non-transfected and stably transfected SH-SY5Y cells with wild-type or mutant *APP* gene

**Figure 1:**
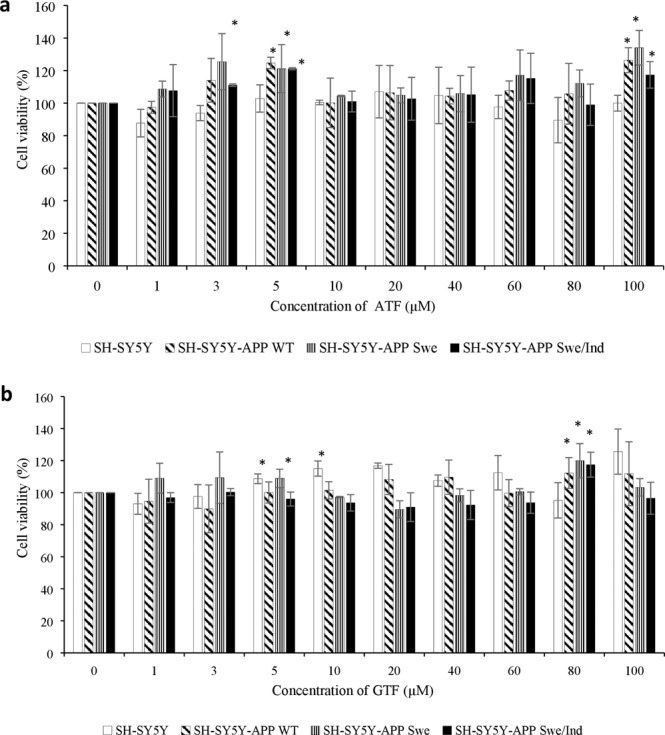
Cytotoxicity effects of ATF and GTF treatment on non-transfected and stably transfected SH-SY5Y cells. *p < 0.05 vs untreated control in each cells group. Data are expressed as means ± SD (N = 3).

To determine the cytotoxicity effect of ATF and GTF, a range of doses from 1 to 100 µM were screened by cell viability assay in non-transfected SH-SY5Y, transfected SH-SY5Y-APP Swe and SH-SY5Y-APP Swe/Ind. Incubation with various concentrations of ATF and GTF for 24 h did not show any significant cytotoxic effect on all cell types (Fig. 1a,b). However, concentrations of 5 µM for each isomer, 100 µM for ATF and 80 µM for GTF showed significant increases in cell viability (p < 0.05) and therefore were chosen for further screening by determining its effect on Aβ-42 production.

### Reduction in Aβ42 level by ATF and GTF in SH-SY5Y cells expressing the *APP* Swe mutation

**Figure 2:**
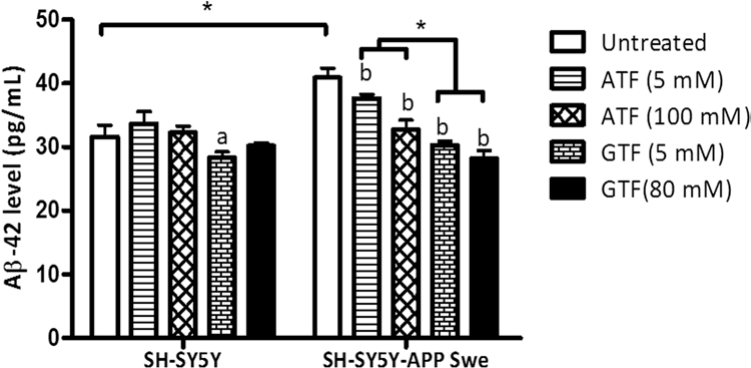
Effects of APP gene mutation and ATF and GTF treatment on Aβ42 level. *significant different between the two treatment groups (p < 0.05), (a) p < 0.05 vs untreated non-transfected SH-SY5Y, (b) p < 0.05 vs untreated SH-SY5Y-APP Swe. Data are expressed as means ± SD (N = 3).

Based on our previous finding, the level of Aβ42 was increased according to the following order: SH-SY5Y < SH-SY5Y-APP WT < SH-SY5Y-APP Swe < SH-SY5Y-APP Swe/Ind^11^. The effect of ATF and GTF on the level of Aβ42 in this study was determined by comparing the non-transfected SH-SY5Y and stably transfected SH-SY5Y-APP Swe as these stably transfected cells represent between minimum and maximum level of Aβ42 in our cell models. At baseline level, there was a significant increase in Aβ42 level in control SH-SY5Y APP Swe as compared to the non-transfected control group (p < 0.05) (Fig. 2). Treatment with ATF or GTF significantly reduced the Aβ42 level in SH-SY5Y-APP Swe compared to its untreated control (p < 0.05) with a significant reduction shown by GTF as compared to ATF for the two doses used. From these observations, both ATF and GTF at selected concentrations could reverse the changes of Aβ42 levels with a higher effect shown by GTF.

### Reduction in mitochondrial ROS level by ATF and GTF in SH-SY5Y cells expressing the mutant *APP* gene

**Figure 3:**
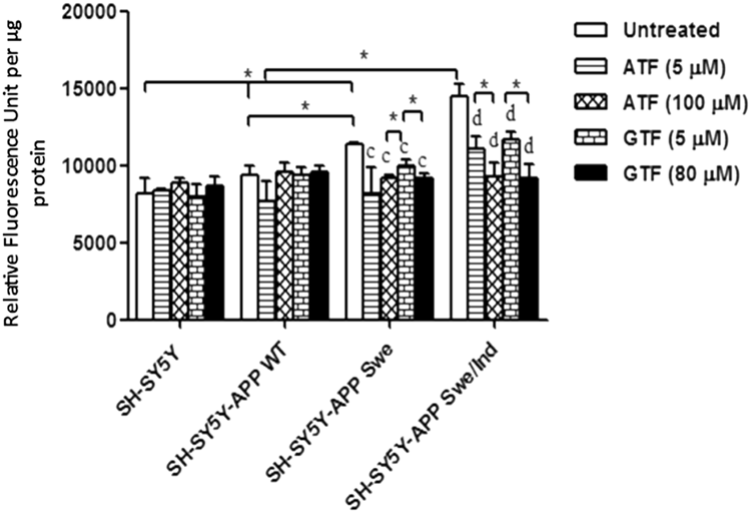
Effects of APP gene mutation and ATF and GTF treatment on mitochondrial ROS level. *significant between the two treatment groups (p < 0.05), (c) p < 0.05 vs untreated SH-SY5Y-APP Swe, (d) p < 0.05 vs untreated SH-SY5Y-APP Swe/Ind. Data are expressed as means ± SD (N = 3).

The baseline level of mitochondrial ROS in SH-SY5Y cells expressing mutated *APP* gene was significantly higher than that in non-transfected cells and cells expressing the WT *APP* gene (Fig. 3) (p < 0.05). SH-SY5Y cells carrying the double-mutant forms of *APP* gene (Swe/Ind) showed significantly higher level of mitochondrial ROS than SH-SY5Y cells carrying the single-mutant form of *APP* gene (Swe) (p < 0.05). Treatment with both ATF and GTF significantly reduced the level of mitochondrial ROS in SH-SY5Y cells carrying the mutated *APP* gene and this effect was higher in cells treated with high concentrations of ATF and GTF (p < 0.05). This showed that both ATF and GTF functioned as antioxidants by reducing the level of ROS.

### Elevation in ATP level by GTF but not ATF treatment in SH-SY5Y cells expressing the mutant *APP* gene

**Figure 4:**
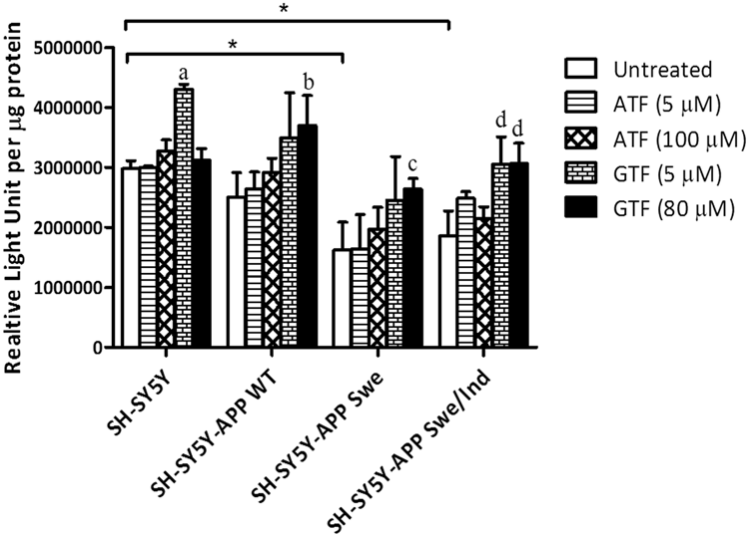
Effects of APP gene mutation and ATF and GTF treatment on ATP level. *significant between the two treatment groups (p < 0.05), (a) p < 0.05 vs untreated non-transfected SH-SY5Y, (b) p < 0.05 vs untreated SH-SY5Y-APP WT, (c) p < 0.05 vs untreated SH-SY5Y-APP Swe, (d) p < 0.05 vs untreated SH-SY5Y-APP Swe/Ind. Data are expressed as means ± SD (N = 3).

At baseline level, the production of ATP was significantly lower in SH-SY5Y expressing mutant *APP* gene compared to non-transfected SH-SY5Y (p < 0.05) (Fig. 4). However, the ATP level was unchanged in SH-SY5Y expressing wild-type gene. After treatment with ATF or GTF, only treatment with GTF at 80 µM significantly increased the level of ATP in all stably transfected cells compared to the untreated group of each cell (p < 0.05) thus indicating the unique role of GTF compared to ATF. A similar significant increase was observed in the ATP level of SH-SY5Y cells carrying the double-mutant form of *APP* gene (Swe/Ind) with GTF treatment at 5 μM (p < 0.05).

### Elevation of complex V activity by GTF or ATF treatment in SH-SY5Y cells expressing the double mutant form of *APP* gene

**Figure 5:**
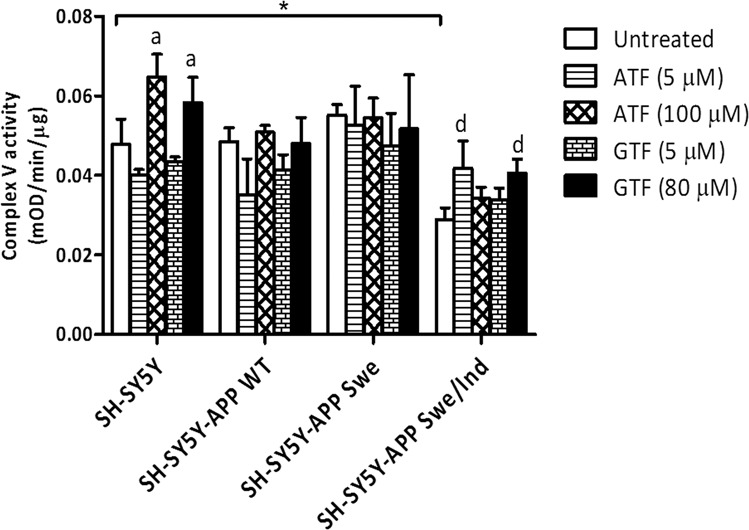
Effects of APP gene mutation and ATF and GTF treatment on complex V enzyme activity. *significant between the two treatment groups (p < 0.05), (a) p < 0.05 vs untreated non-transfected SH-SY5Y, (d) p < 0.05 vs untreated SH-SY5Y-APP Swe/Ind. Data are expressed as means ± SD (N = 3).

Complex V activity was significantly reduced in the group of SH-SY5Y-cells expressing the double mutant form of *APP* gene (Swe/Ind) compared to the non-transfected SH-SY5Y control cells (p < 0.05) (Fig. 5). Treatment with ATF (100 uM) and GTF (80 uM) significantly increased complex V enzyme activity in non-transfected SH-SY5Y cell group compared to the untreated control group (p < 0.05). Similarly, treatment with ATF (5 μM) and GTF (80 μM) significantly increased complex V enzyme activity in SH-SY5Y-APP Swe/Ind cell group compared to the untreated control group of SH-SY5Y cells (p < 0.05).

### Differential effect of ATF and GTF on the expression of genes involved in mitochondrial membrane permeability regulation

**Figure 6:**
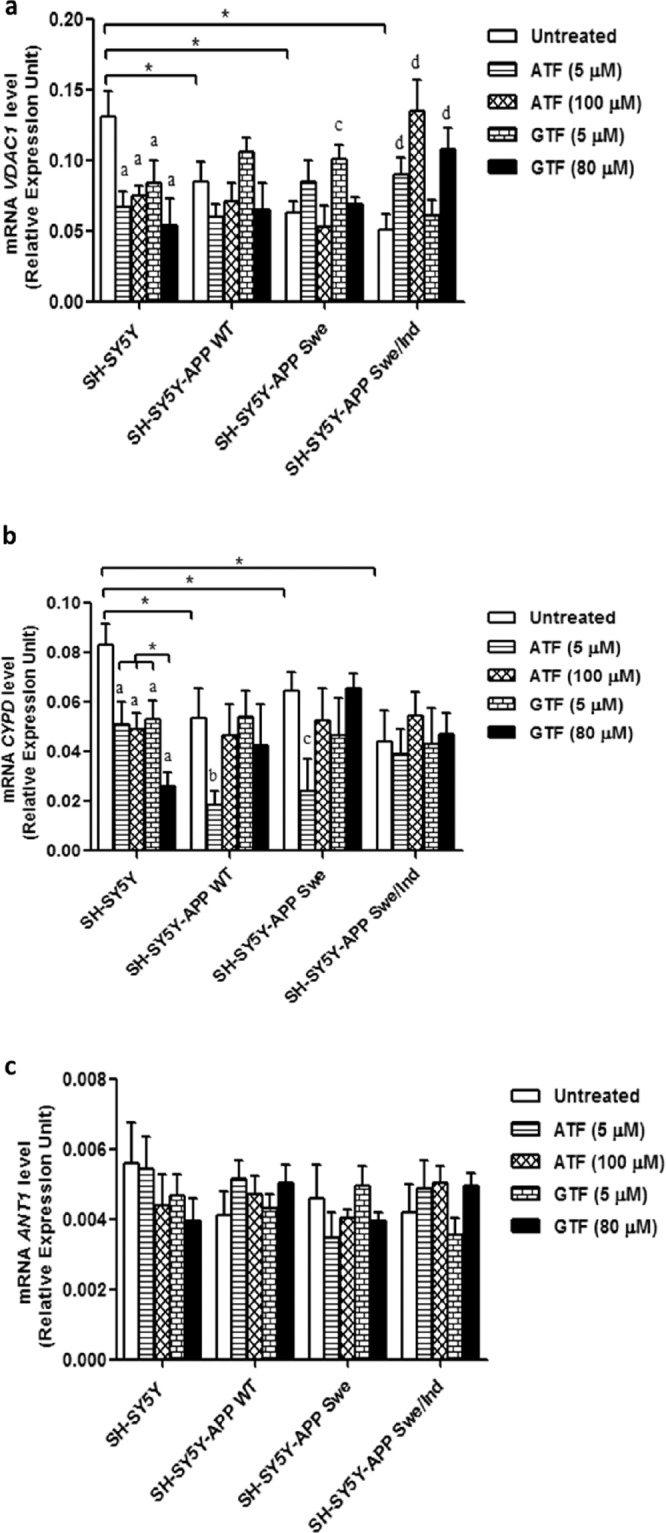
Effects of APP gene mutation and ATF and GTF treatment on genes expression involved in mPTP regulation. (**a**) *VDAC1*, (**b**) *CYPD* and **(c)** *ANT*. *significant between the two treatment groups (p < 0.05), (a) p < 0.05 vs untreated non-transfected SH-SY5Y, (b) p < 0.05 vs untreated SH-SY5Y-APP WT, (c) p < 0.05 vs untreated SH-SY5Y-APP Swe, (d) p < 0.05 vs untreated SH-SY5Y-APP Swe/Ind. Data are expressed as means ± SD (N = 3).

The baseline expression levels of *VDAC1* and *CYPD* were significantly reduced in all stably transfected cells as compared to the non-transfected control (p < 0.05) (Fig. 6a,b). However, no significant difference was observed in the baseline expression level of *ANT* gene between all groups (Fig. 6c). Treatment with both ATF and GTF significantly reduced the expression of *VDAC1* and *CYPD* in non-transfected SH-SY5Y cells as compared to the untreated control (p < 0.05). In cells transfected with Swe/Ind double-mutant, treatment with ATF and GTF (80 µM) significantly increased *VDAC1* gene expression (p < 0.05). On the other hand, treatment of SH-SY5Y cells expressing WT or Swe mutant with 5 µM of ATF resulted in a significant decrease in *CYPD* gene expression (p < 0.05). These observations showed that both ATF and GTF differentially affect the mRNA expression of *VDAC1* and *CypD* in cells with different types of APP mutation.

### Reduction in GTF-mediated cyclophilin D (CypD) protein expression in SH-SY5Y cells stably transfected with WT or mutant *APP* gene

**Figure 7:**
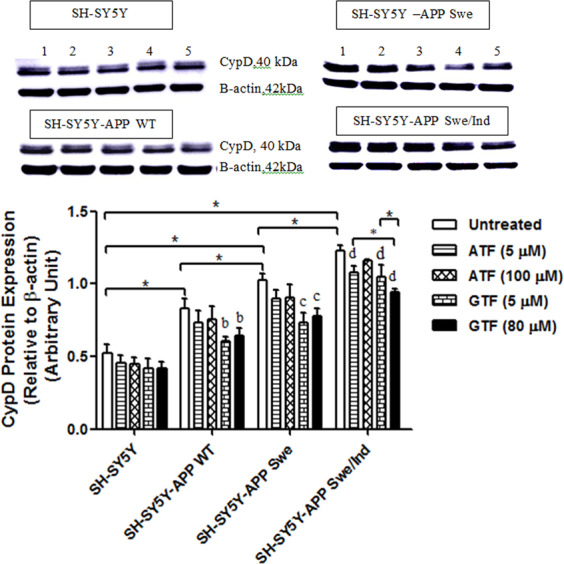
Effects of APP gene mutation and ATF and GTF treatment on CypD protein expression. Lane 1: untreated cells, Lane 2: ATF-treated cells (5 µM), Lane 3: ATF-treated cells (100 µM), Lane 4: GTF-treated cells (5 µM), Lane 5: GTF-treated cells (80 µM) (The full image of the membrane was shown in supplementary file). *significant between the two treatment groups (p < 0.05), (b) p < 0.05 vs untreated SH-SY5Y-APP WT, (c) p < 0.05 vs untreated SH-SY5Y-APP Swe, (d) p < 0.05 vs untreated SH-SY5Y-APP Swe/Ind. Data are expressed as means ± SD (N = 3).

To further elucidate the effect of ATF and GTF on mitochondrial membrane permeability, the expression level of key protein CypD was determined. The baseline expression level of CypD was significantly increased in the following order: non-transfected SH-SY5Y < SH-SY5Y-APP WT < SH-SY5Y-APP Swe < SH-SY5Y-APP Swe/Ind (p < 0.05). (Fig. 7). Treatment with GTF at 5 and 80 µM concentrations significantly decreased the CypD protein expression in all stably transfected SH-SY5Y cells as compared to their respective untreated controls (p < 0.05). No decrease was observed for CypD expression after ATF treatment suggesting an effect of GTF that was not shared with ATF.

### Reduction in cytochrome c release by ATF and GTF in SH-SY5Y cells expressing the mutant *APP* gene

**Figure 8:**
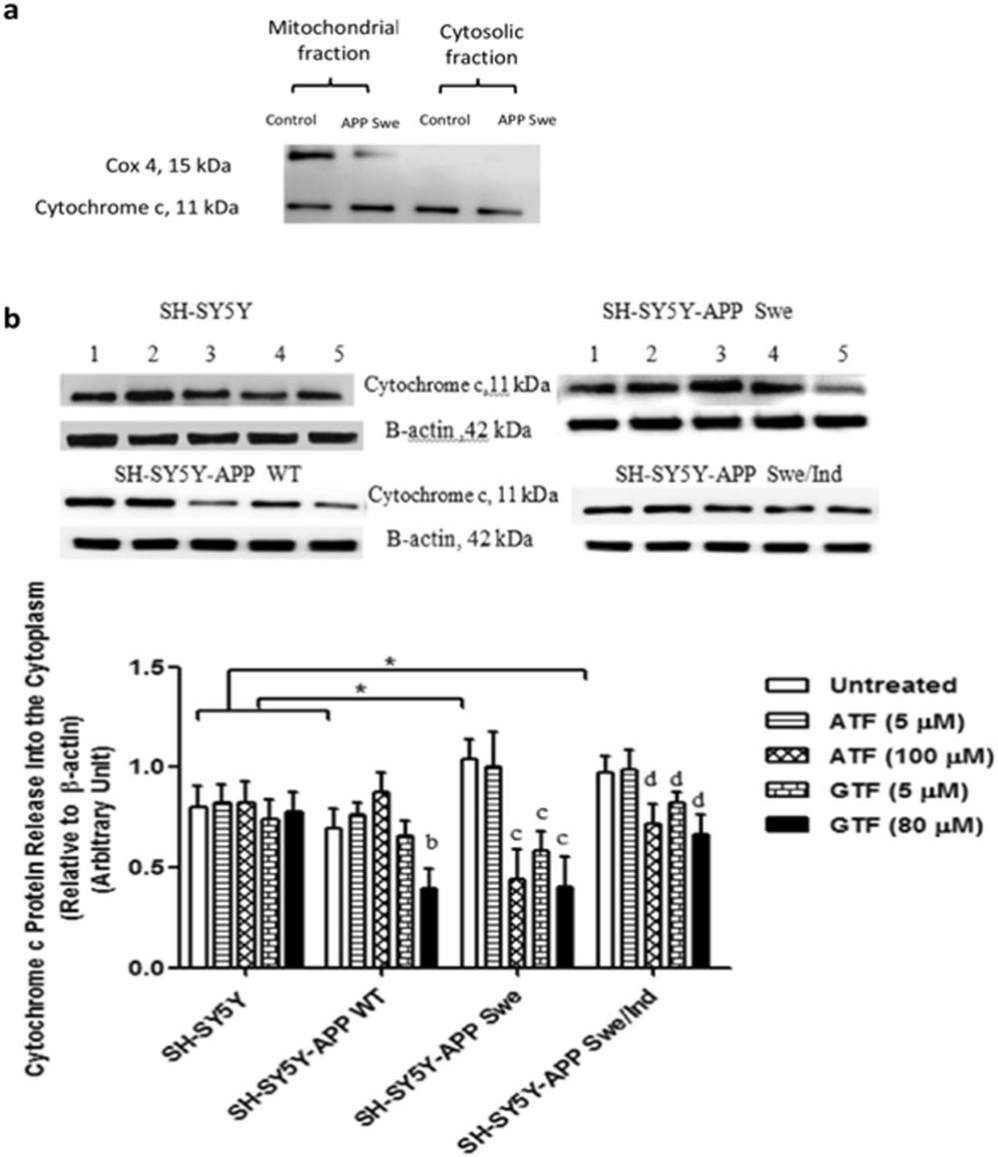
Effects of APP gene mutation and ATF and GTF treatment on cytochrome c release. Lane 1: untreated cells, Lane 2: ATF-treated cells (5 µM), Lane 3: ATF-treated cells (100 µM), Lane 4: GTF-treated cells (5 µM), Lane 5: GTF-treated cells (80 µM) (The full image of the membrane was shown in supplementary file). *significant between the two treatment groups (p < 0.05), (b) p < 0.05 vs untreated SH-SY5Y-APP WT, (c) p < 0.05 vs untreated SH-SY5Y-APP Swe, (d) p < 0.05 vs untreated SH-SY5Y-APP Swe/Ind. Data are expressed as means ± SD (N = 3).

The absence of Cox 4 protein confirmed the purity of cytoplasmic fraction from contamination with mitochondrial protein (Fig. 8a). Analysis of the baseline expression level of cytochrome c release showed that SH-SY5Y cells stably transfected with the mutant *APP* gene significantly increased the release of cytochrome c in the cytoplasm as compared to WT *APP*-expressing cells and non-transfected SH-SY5Y cells (p < 0.05) (Fig. 8b). Treatment with high concentrations of ATF (100 µM) and GTF (5 and 80 µM) significantly reduced the release of cytochrome c into the cytoplasm of SH-SY5Y cells stably expressing the mutant *APP* as compared to the untreated control (p < 0.05). In the WT *APP*-expressing cells, a similar significant reduction in cytochrome c release was observed with 80 µM of GTF treatment (p < 0.05).

### Reduction in BAX/Bcl-2 ratio by ATF and GTF in SH-SY5Y cells stably transfected with the WT and mutant *APP* gene

**Figure 9:**
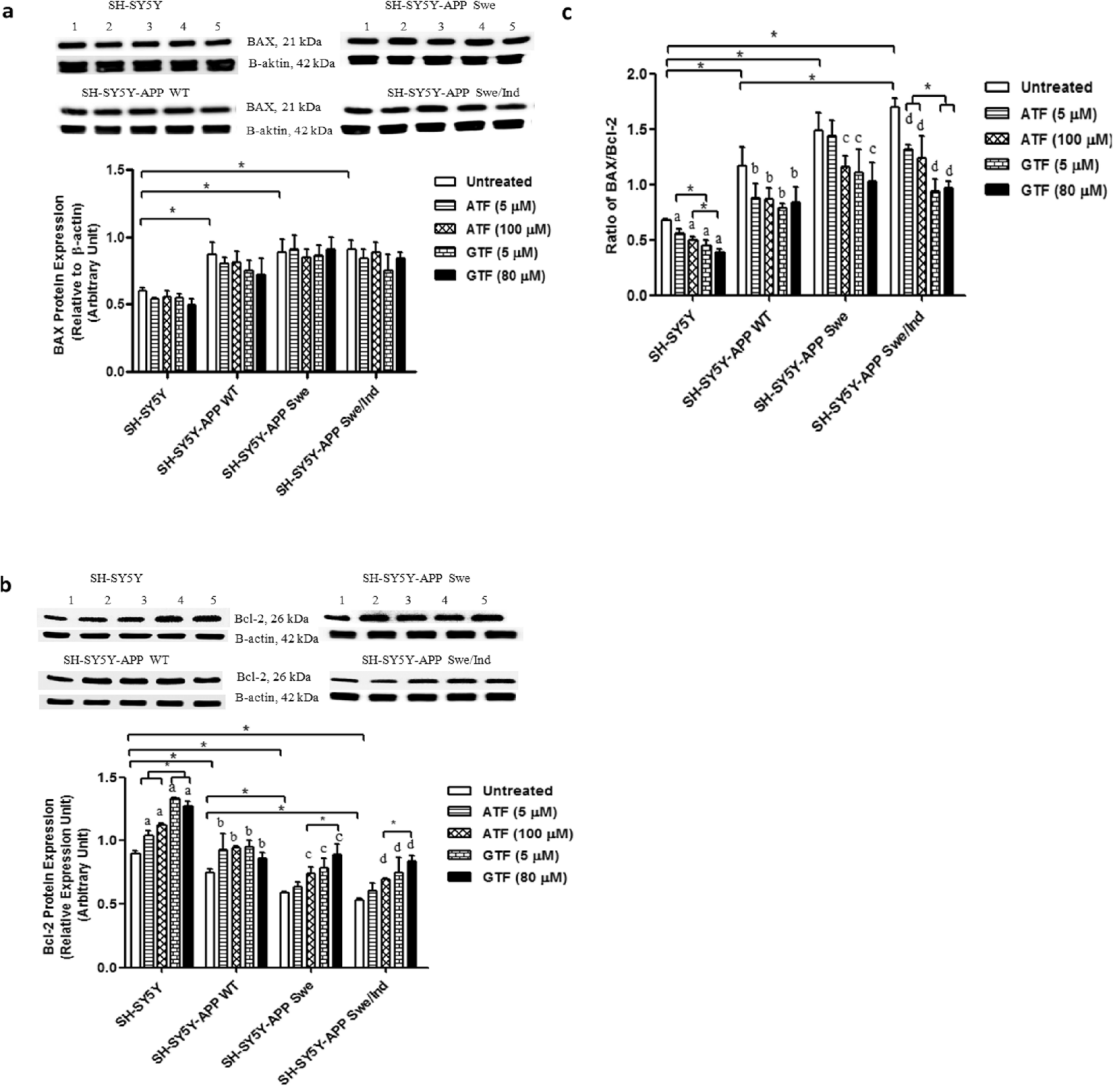
Effects of APP gene mutation and ATF and GTF treatment on protein expression of **(a)** BAX, **(b)** Bcl-2 and **(c)** the ratio of BAX/Bcl-2. Lane 1: untreated cells, Lane 2: ATF-treated cells (5 µM), Lane 3: ATF-treated cells (100 µM), Lane 4: GTF-treated cells (5 µM), Lane 5: GTF-treated cells (80 µM) (The full image of the membrane was shown in supplementary file). *significant between the two treatment groups (p < 0.05), (a) p < 0.05 vs untreated non-transfected SH-SY5Y, (b) p < 0.05 vs untreated SH-SY5Y-APP WT, (c) p < 0.05 vs untreated SH-SY5Y-APP Swe, (d) p < 0.05 vs untreated SH-SY5Y-APP Swe/Ind. Data are expressed as means ± SD (N = 3).

The baseline expression level of the pro-apoptotic protein BAX was significantly increased in all cells stably transfected with the WT or mutant *APP* gene than the non-transfected control (p < 0.05) (Fig. 9a). However, no significant difference was observed following ATF and GTF treatments in all SH-SY5Y cell types. On the contrary, the baseline expression level of the anti-apoptotic protein, Bcl-2, was significantly decreased in all stably transfected SH-SY5Y cells, with a significant reduction observed in SH-SY5Y cells carrying *APP* gene mutation than those carrying the WT mutant as well as the non-transfected SH-SY5Y cells (p < 0.05) (Fig. 9b). The treatments with ATF (100 µM) and GTF (5 and 80 µM) significantly increased Bcl-2 protein expression in all types of SH-SY5Y cells as compared to their respective untreated controls (p < 0.05).

The ratio of BAX/Bcl-2 was increased in all stably transfected SH-SY5Y cells, with a significant increase observed in SH-SY5Y cells carrying *APP* gene mutation as compared to the WT *APP* gene-expressing cells and non-transfected cells (p < 0.05) (Fig. 9c). The treatments effect on the ratio of BAX/Bcl-2 was similar to that on Bcl-2 protein expression level. GTF exerted stronger effects on the ratio of BAX/Bcl-2 in SH-SY5Y-APP Swe/Ind cells compared to ATF (p < 0.05).

### Reduction in pro-apoptotic pro-caspase-3 protein expression by only GTF treatment in cells stably transfected with the WT or mutant APP gene

**Figure 10:**
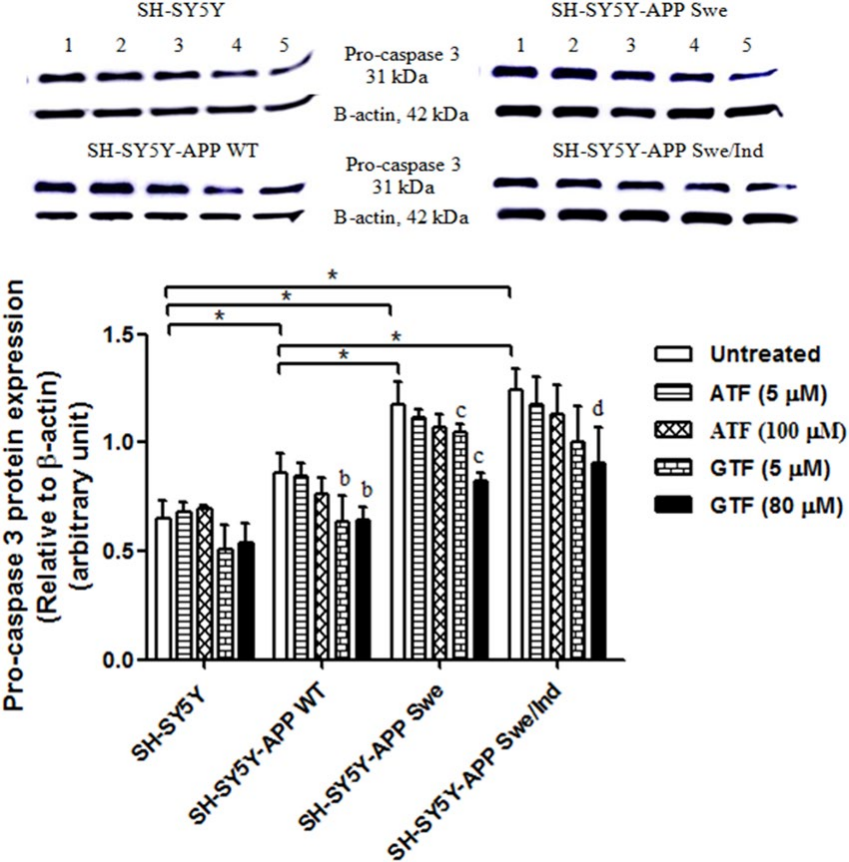
Effects of APP gene mutation and ATF and GTF treatment on the expression of pro-apoptotic protein pro-caspase-3. Lane 1: untreated cells, Lane 2: ATF-treated cells (5 µM), Lane 3: ATF-treated cells (100 µM), Lane 4: GTF-treated cells (5 µM), Lane 5: GTF-treated cells (80 µM). (The full image of the membrane was shown in the supplementary file). *significant between the two treatment groups (p < 0.05), (b) p < 0.05 vs untreated SH-SY5Y-APP WT, (c) p < 0.05 vs untreated SH-SY5Y-APP Swe, (d) p < 0.05 vs untreated SH-SY5Y-APP Swe/Ind. Data are expressed as means ± SD (N = 3).

The baseline expression level of the pro-apoptotic protein pro-caspase-3 was significantly increased in all SH-SY5Y cells stably transfected with the WT or mutant *APP* gene than in the non-transfected control (p < 0.05) (Fig. 10). However, the cells carrying the mutant *APP* gene showed significantly higher expression of pro-caspase-3 than those stably transfected with the WT *APP* gene (p < 0.05). Only treatment with GTF significantly decreased the expression of pro-caspase-3 in all SH-SY5Y cells stably transfected with the WT or mutant *APP* gene than their respective untreated controls (p < 0.05) suggesting an effect of GTF not shared with ATF.

## Discussion

**Figure 11:**
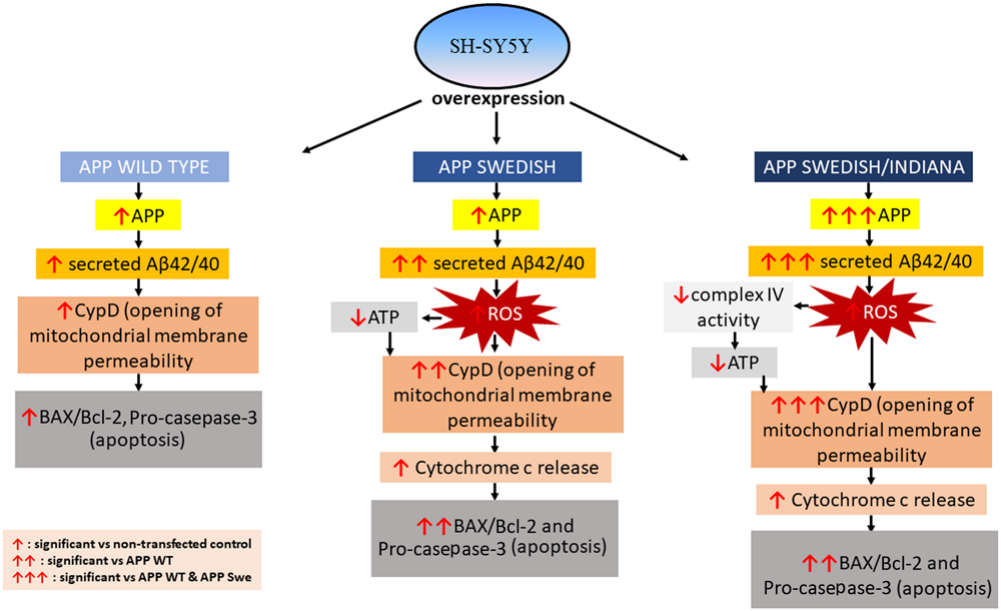
The schematic diagram showing the changes in the mitochondrial function and apoptosis proteins marker in cells expressing different types of *APP* gene mutation.

APP plays a critical role in the pathophysiology of AD^21^ (Zheng & Khoo 2011). Proteolysis of this precursor molecule generates a polypeptide, Aβ which is a primary component of the amyloid plague found in AD patient’s brain. In particular, this Aβ was shown to localize in the mitochondria and transported into this organelle through translocase of the outer mitochondrial membrane protein^22^ (Pinho *et al*. 2014). This further postulated that mitochondria dysfunction plays a critical role in the pathophysiology and alterations in AD. In the present study, we found that the different types of *APP* gene mutation exerted different degree of changes on mitochondrial function and apoptosis proteins marker (Fig. 11). Mutant *APP* gene caused significant changes to the mitochondrial function as shown by  changes in mitochondrial ROS level, ATP level and complex V enzyme activity.

Based on our previous study, these groups of cells demonstrated increased Aβ42 level in the following order: SH-SY5Y cells transfected with the double-mutant (Swe/Ind) *APP* gene > single mutant (Swe) *APP* gene > WT *APP* gene^11^. AD has been reported to be linked to increased oxidative stress due to increased production of ROS and RNS and impairment of antioxidant defense enzymes functions^23,24^. Previous study on primary cultures of mouse cerebral cortical neurons treated with Aβ42 showed that the level of intracellular ROS was increased with Aβ42 treatment accompanied with mitochondrial dysfunction^25,26^.

Vitamin E is one of the hydrophilic antioxidants which acts as an oxidation chain breaker in lipid environment. A previous study showed that vitamin E significantly reduced the ROS level by efficiently scavenge the peroxyl radical (ROO•)^27^ (Niki 2014). Current vitamin E research however, showed increase evidence that indicates vitamin E can act as a non-antioxidant to exert its biological modulation. Our findings showed that ATF and GTF at selected doses increased cell viability and decreased mitochondrial ROS level. This finding is supported by a previous study, wherein ATF reduced ROS production in primary rat embryonic hippocampal neuronal cells induced by Aβ42^2^. Both isoforms of vitamin E, ATF and GTF showed similar effects as an antioxidant in reducing mitochondrial ROS. However, high concentrations of both ATF and GTF exerted more pronounced effects than low concentrations. Interestingly, high concentration GTF was able to reduce ROS in cell carrying both type of APP gene mutation including Swe and Swe/Ind. However, very few studies have focused on the effect of GTF on ROS production in an AD model even though GTF had been shown to reduce oxidative stress related to reactive nitrogen species in the brain^5^.

Through the non-antioxidant activity of vitamin E, our present study also showed both ATF and GTF reduced the level of Aβ42. A previous study reported that depletion of vitamin E in a mouse model of AD has been shown to increase Aβ accumulation by decreasing its clearance from the brain and blood^28^. Findings from another study showed that neurotoxicity of Aβ42 can effectively be ameliorated by the administration of vitamin E^29^. Several genes involved in the clearance of Aβ protein were strongly affected in the hippocampus of male albino rats fed with dietary vitamin E that mainly comprises GTF, indicating the protective effect of GTF on AD progression^6^. Based on these data, it is suggested that both ATF and GTF play a significant role in regulating Aβ level. However, our data showed that GTF reduced Aβ42 at a greater level as compared to ATF.

The Aβ42 peptide was found to interact with many proteins in mitochondria^22^ (Pinho *et al*. 2014). Furthermore, the interaction of Aβ was not limited to proteins located at the inner mitochondrial membrane but also with proteins located at the outer mitochondrial membrane. Binding of Aβ to CypD, a master regulator of mitochondrial membrane permeability activates the opening of the membrane uncontrollably that later lead to mitochondrial swelling^13^. Our findings showed decreased *CypD* gene expression which may reflect the negative feedback regulation as CypD protein expression was increased. Therefore, accumulation of the Aβ42 in the mitochondria induced mitochondrial dysfunction and later lead to the higher mitochondrial ROS production^13^. Between treatment of ATF and GTF, only 80 µM GTF treatment showed a significant reduction in the CypD protein expression in all transfected SH-SY5Y cells. As GTF could reduce the level of Aβ greatly, there would be less interaction between CypD and Aβ. While very few studies have evaluated the direct effect of GTF on CypD and Aβ protein expression, a previous study had shown that walnut extract rich in GTF potentially inhibits the fibrillization of Aβ^30^ and defibrillizes the preformed fibrils^31^, which may prevent the binding of Aβ to CypD.

One of the most prominent mitochondrial dysfunctions caused by Aβ accumulation was reduction in ATP production. ATP synthase or complex V is the enzyme in the mitochondria responsible for ATP production. Aβ has been shown to be similar in structure to the ATP synthase binding sequence of the inhibitor of F1 (IF_1_), a naturally occurring inhibitor of complex V in the mitochondria. The binding of Aβ to α-subunit of F_1_F_0_ ATP synthase, hindering this enzyme to bind to its substrate thus inhibits its activity and eventually lead to the reduction in ATP production^20^. This is in agreement to our finding where the reduction in complex V activity was accompanied by a reduction in ATP level in SH-SY5Y-APP Swe/Ind cells. Transgenic mice carrying double *APP* mutation (Swedish and London) also showed a similar pattern of inhibition whereby the mitochondrial membrane potential, ATP level, and complex V activity were decreased at the early age of 3 months old^32^. In the present study, only GTF was able to reverse ATP level and complex V activity in stably transfected SH-SY5Y cells expressing mutant *APP* gene. As mentioned above, GTF significantly reduced the level of Aβ, and this might be the reason as to why only GTF was observed to improve mitochondrial function parameters such as complex V activity and ATP level. In addition, the presence of high amount of Aβ could inhibit complex V enzyme activity by binding to the active subunit of the complex thus hindering its catalytic activity to convert ADP to ATP^20^.

The increase in mitochondrial membrane permeability which is regulated mainly by CypD protein will lead to the release of cytochrome c into the cytoplasm and the induction of apoptosis^33,34^. This event could be triggered by increased mitochondrial ROS level and mitochondrial membrane permeability as well as decreased ATP level as observed in this study. We have shown that decreased level of CypD protein expression after GTF treatment was also accompanied with decreased cytochrome c release into the cytoplasm. There is limited evidence to show the direct interaction between GTF and cytochrome c. However, cytochrome c could exit mitochondria if there is an increased mitochondrial membrane permeability primarily regulated by CypD protein^35^. Therefore, the regulation of CypD protein expression by GTF might also regulate the cytochrome c release which was not shown by ATF.

**Figure 12:**
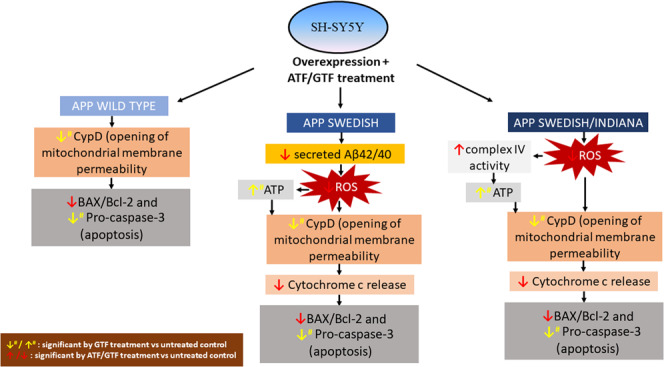
The schematic diagram showing the effect of ATF or GTF treatment on the mitochondrial function and apoptosis proteins marker in cells expressing different types of *APP* gene mutation.

The improvement of mitochondrial function with GTF treatment observed in the present study was also accompanied with the reduction in the expression of apoptosis protein marker pro-caspase-3 that was not shown with ATF treatment (Fig. 12). The ratio of BAX/Bcl-2 and expression of pro-caspase-3 were high in SH-SY5Y-APP Swe/Ind cells indicating increased of apoptosis event which was in accordance with the degree of *APP* gene mutation. Treatment with ATF at 100 µM and GTF at 5 and 80 µM decreased the ratio of BAX/Bcl-2 in mutant SH-SY5Y cells indicating inhibition of apoptosis. However, the effect of GTF in decreasing  the ratio of BAX/Bcl-2 in SH-SY5Y cells carrying double-mutant *APP* was higher than that of ATF. In addition, only GTF at 80 µM was shown to decrease the expression of pro-caspase-3 in all transfected SH-SY5Y cells. The anti-apoptotic properties of ATF related to the decreased ratio of BAX/Bcl-2 was demonstrated in a study using cortical neurons exposed to hydrogen peroxide^36^. Studies on anti-apoptotic effects of GTF in AD are limited, as most reports emphasized its role in promoting death of cancer cells^37,38^. A previous study showed that GTF failed to exert any cytotoxic effects on normal cells, suggesting that GTF may be effective in the regulation of apoptosis pathway depending on the cell type^39^. In addition, GTF showed better neuroprotective effects than ATF by preventing the cytotoxicity of methyl-4-phenyl-1, 2, 3, 6-tetrahydropyridine (MPTP) in Parkinson’s disease^40^. In summary, GTF showed better performance than ATF in improving mitochondrial functions and prevent the apoptosis event by acting as a stronger antioxidant and non antioxidant  thus, prevent Aβ accumulation.

Nevertheless, limitations in this study may include the use of SH-SY5Y cells which were undifferentiated and have cancer-like properties thus not mimicking post-mitotic neurons. However, these cells are commonly used to study the pathology of AD and the untreated control group was used to compare any changes that occur resulted from overexpression of APP with or without mutation and treatment with ATF or GTF. The current findings were based from an *in vitro* study involving a type of cell to elucidate specific biochemical processes thus, limiting the effect of ATF and GTF on the complexity of the disease process that can be investigated *in vivo*. Another limitation is the usage of the housekeeping gene in this study. In particular, GAPDH has been widely used as a housekeeping gene and control marker for cytosolic protein expression. However, it has been reported as a controversial marker in AD research. A previous study showed that GAPDH protein interact with Aβ which altered the activity of this protein^41^(Mazzola & Michael 2001). Nevertheless, a similar study showed that the gene expression of GAPDH remained unchanged regardless of different concentrations of Aβ treatment thus, supporting the fact that GAPDH can be used for the gene expression studies and not affected by the transfection of *APP* gene in the induction of the AD model. Moreover, in this study comparisons were carried out between the treatments of ATF and GTF with their respective controls.

Although the present study showed that both ATF and GTF have beneficial effects in regulating mitochondrial function and apoptosis proteins marker expression, there were several evidences that showed non-beneficial effect of vitamin E. A study by Grimm *et al*.^42^ showed that tocopherol isomers (α, γ, δ) at 10 µM increased the production and decreased the degradation of Aβ in SH-SY5Y over expressing wild type APP by increasing the protein expression of β- and γ-secretase enzyme. However, their results showed that ATF had only minor effects on Aβ production whereas delta-tocopherol (DTF) has the highest potency to increase Aβ generation. Besides acting as an antioxidant, vitamin E also has been reported to act as a pro-oxidant especially when present at high doses^43^. At high concentrations of vitamin E, peroxidase is oxidized to phenoxyl radicals to induce microsomal lipid peroxidation^44^. The activity of antioxidant and pro-oxidant of vitamin E was shown to be influenced by the concentration of co-antioxidant such as an ascorbate and the oxidative environment. In the presence of high concentrations of co-antioxidants and under mild oxidative conditions, α-tocopherol normally behave as an antioxidant^45^. Despite being well-known as an antioxidant, targeting oxidative defenses so far did not change the course of cognitive decline in AD and thus reflecting the role of vitamin E as antioxidant paradox^46,47^. Even though there are many conflicting reports of positive and negative results of vitamin E on the same biological activities in the literature that contribute to the inconclusiveness, several literatures appear to support the view that the benefits outweigh the side-effects of vitamin E^48,49^. In addition, the different isomers of vitamin E have been reported to exert different biological effects.

## Conclusions

Both ATF and GTF act as antioxidants and anti-amyloid by reducing the mitochondrial ROS and Aβ42 level in SH-SY5Y cells expressing mutated *APP* gene. However, the anti-amyloid effect of GTF is greater than ATF suggesting its potential in improving the mitochondrial function by increasing the ATP level and complex V enzyme activity. It also reduced the mitochondrial membrane permeability as indicated by the reduced expression of CypD protein which later lead to the prevention of apoptosis event as shown by the reduction in pro-caspase-3 protein expression which was not seen with ATF treatment. Thus, these findings may add to its potential role in the development of therapeutic agents for AD.

## Supplementary information

Supplementary Dataset 1.

## Data Availability

The data generated during this study is included in this published article or available upon reasonable request.
