# Supplementary material for: Comparative Effects of Alpha- and Gamma-Tocopherol on Mitochondrial Functions in Alzheimer’s Disease In Vitro Model
Source: Sci Rep. 2020 Jun 2;10:8962. doi: 10.1038/s41598-020-65570-4 (PMC7265356; doi:10.1038/s41598-020-65570-4)

Comparative Effects of Alpha- and Gamma-Tocopherol on Mitochondrial Functions in Alzheimer's Disease In Vitro Model

Aslina Pahrudin Arrozi[1], Siti Nur Syazwani Shukri[1], Wan Zurinah Wan Ngah[1], Yasmin Anum Mohd Yusof[1], Mohd Hanafi Ahmad Damanhuri[1], Faizul Jaafar[1], Suzana Makpol[1,*]

[1]Department of Biochemistry, Level 17, Preclinical Building, Universiti Kebangsaan Malaysia Medical Centre, Jalan Yaacob Latif, Bandar Tun Razak, Cheras, 56000 Kuala Lumpur, Malaysia

Figure Legends
1: Untreated control
2: 5 µM ATF
3: 100 µM ATF
4: 5 µM GTF
5: 80 µM GTF
Representative images

Full Image of Western blotting

Optimization of Pro-caspase-3 using different sample

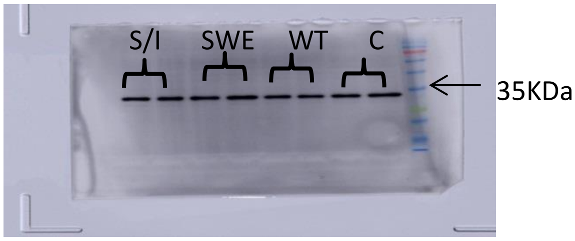

Optimization of β-actin using different sample

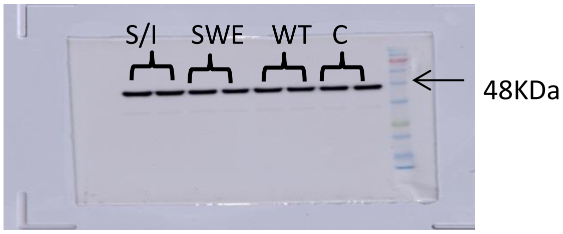

Pro-caspase-3 (pro-caspase 3 and β-actin were detected on the same membrane but one after another)

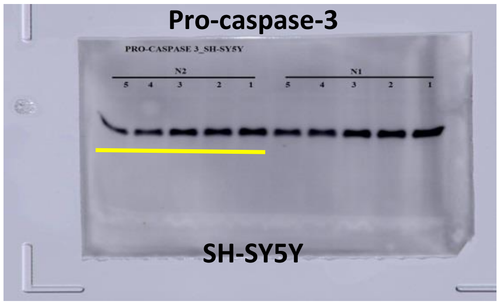

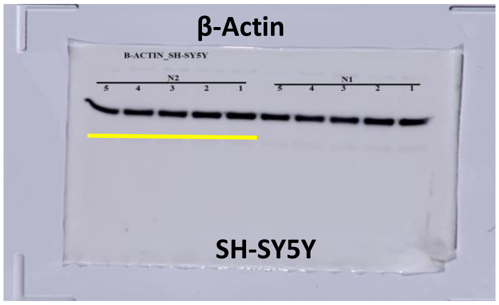

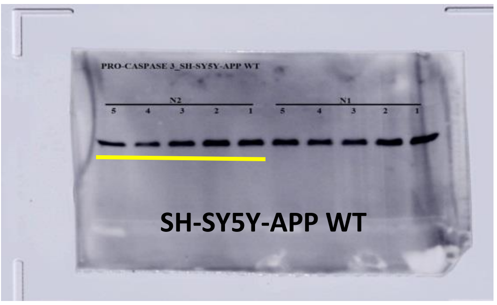

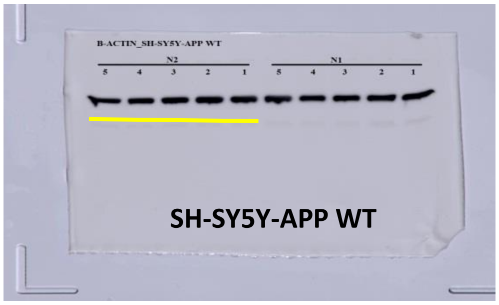

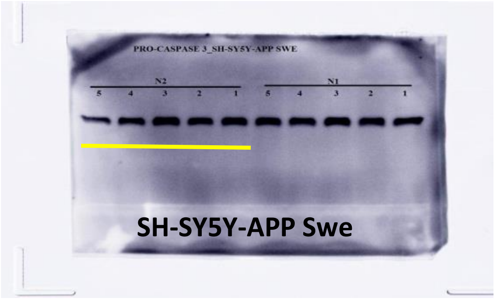

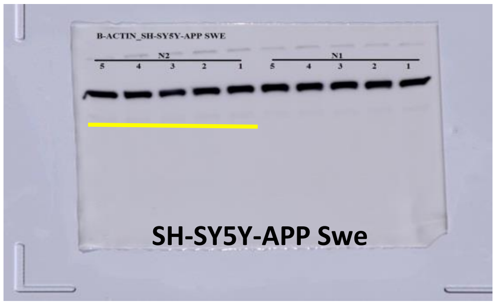

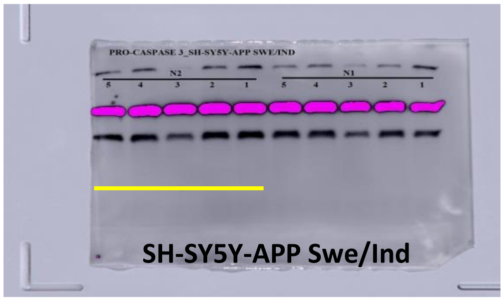

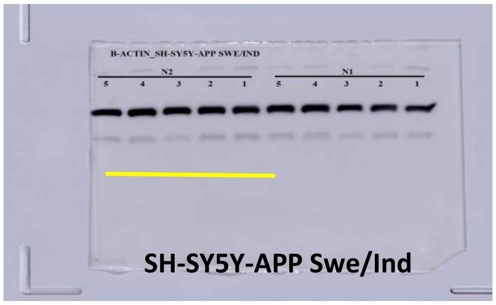

Simultaneous detection of pro-caspase-3 and β-actin, resulted in overexposure of β-actin, as shown by the purple band. Because of this reason, pro-caspase-3 and β-actin were detected at different time and one after another. This apply to all other proteins.

Bcl-2 (Bcl-2 and β-actin were detected on the same membrane but one after another)

Optimization of BAX and Bcl-2 using different sample

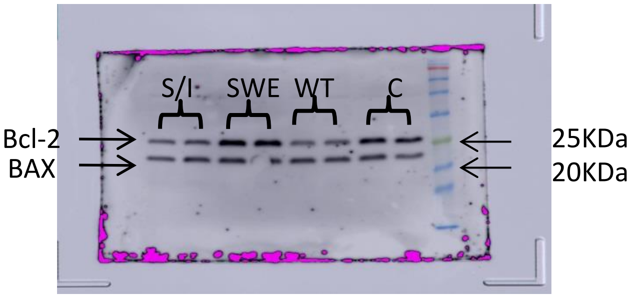

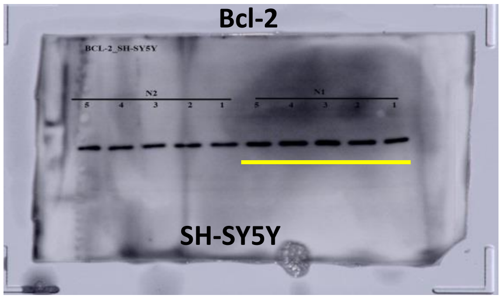

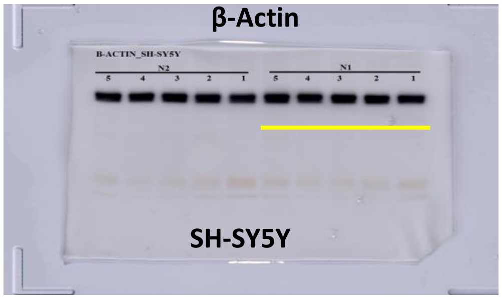

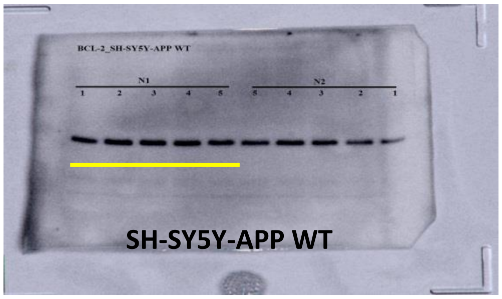

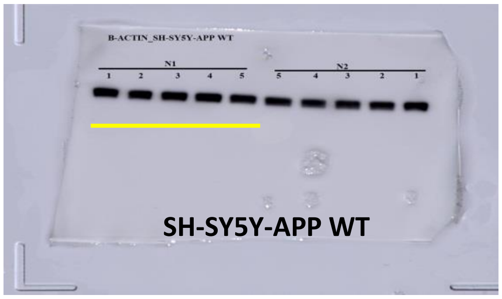

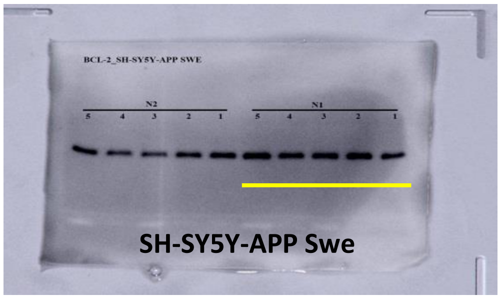

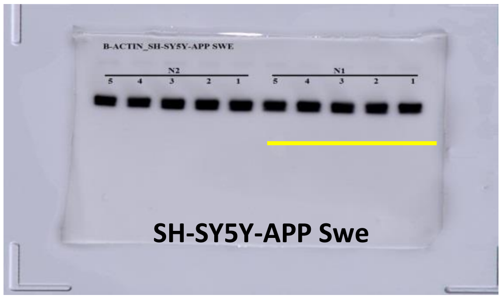

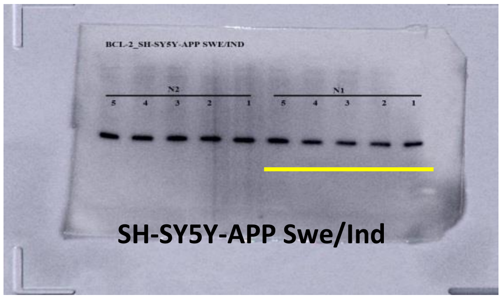

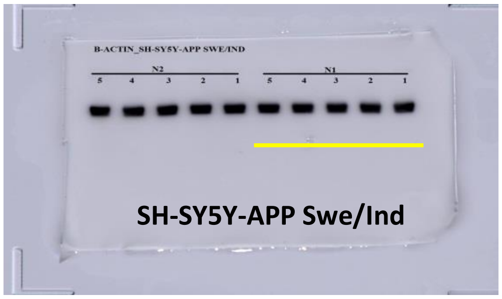

BAX (BAX and β-actin were detected on the same membrane but one after another)

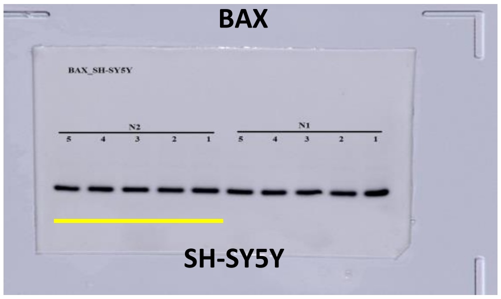

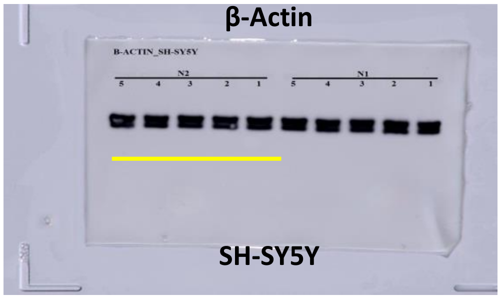

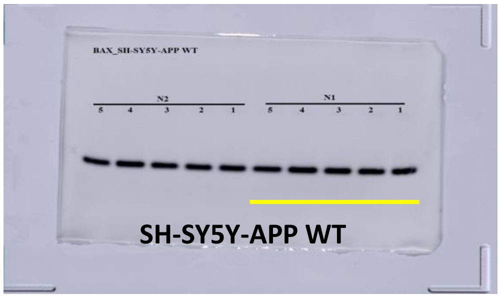

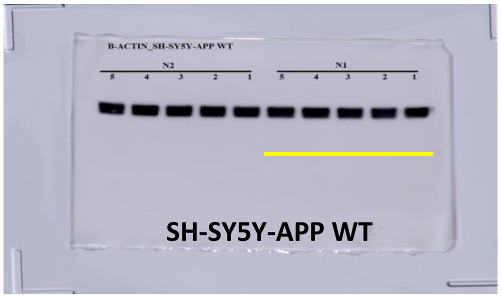

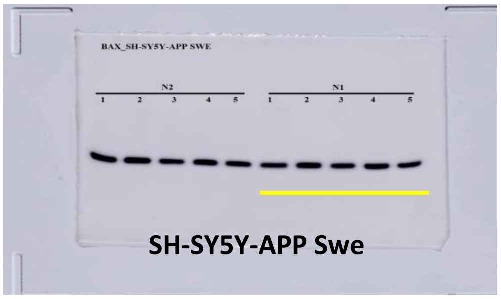

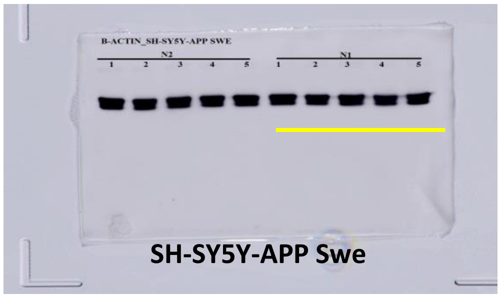

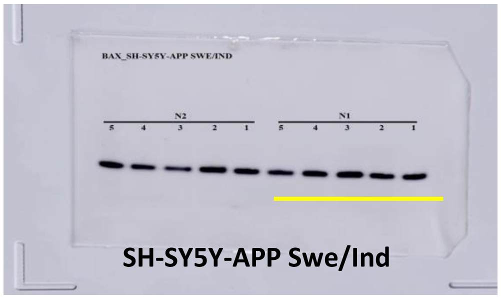

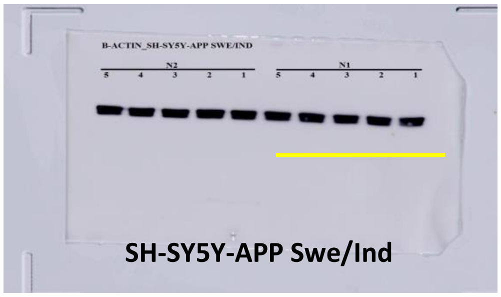

Cytochrome C

The membrane was cut into half for the detection of β-actin (upper part) and cytochrome c (lower part)

Cytochrome C **β-Actin**

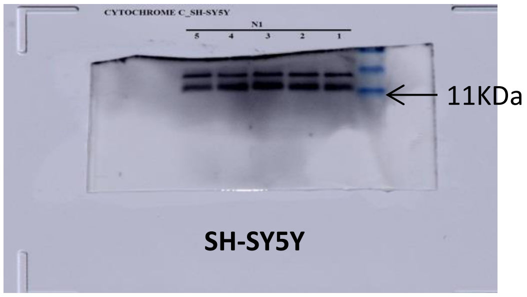

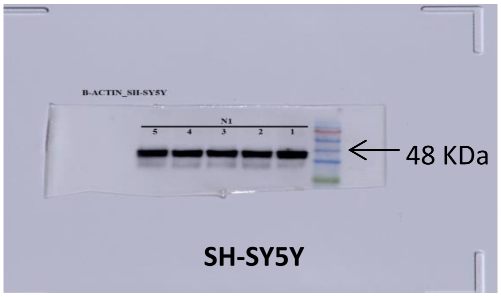

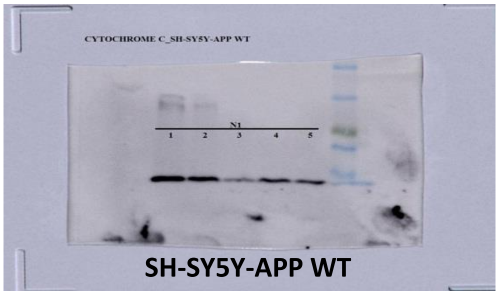

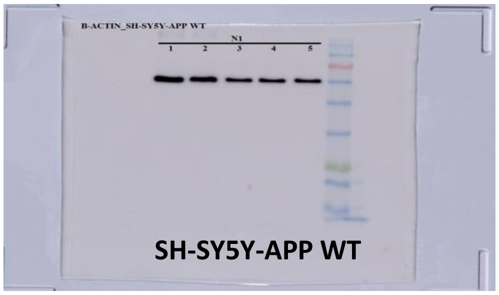

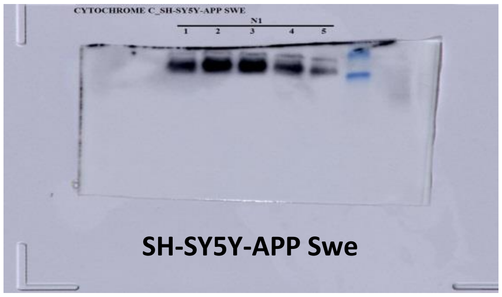

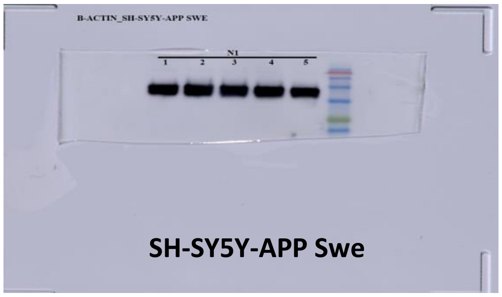

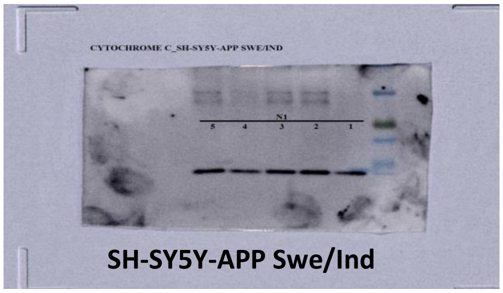

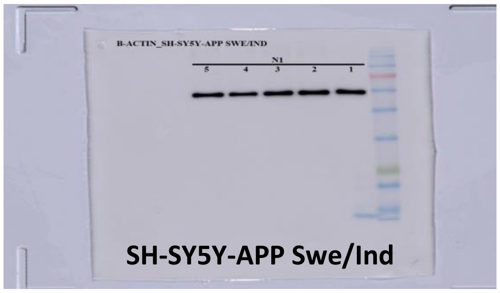

CYPD

The molecular weight of CYPD and β-actin was almost the same (CYPD: 40kDa, β-actin: 42kDa), therefore all samples were run on 2 gels, 1 gel to detect CYPD and the other to detect β-actin.

CYPD | **β-Actin**

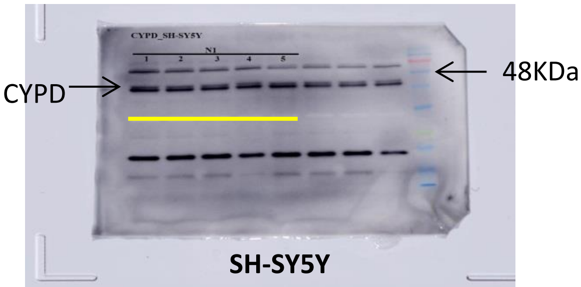

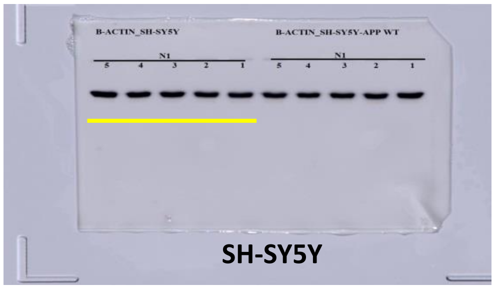

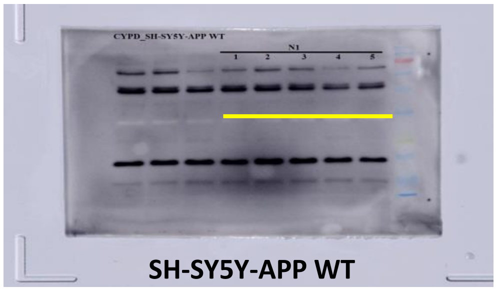

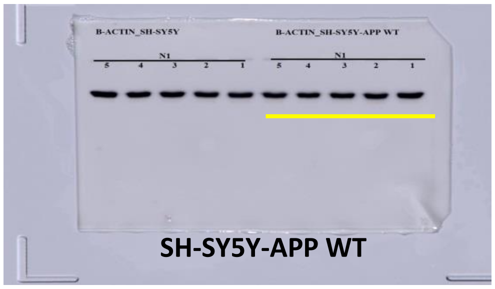

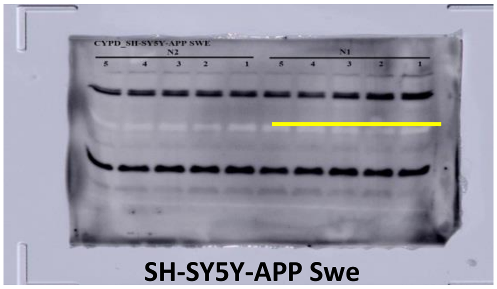

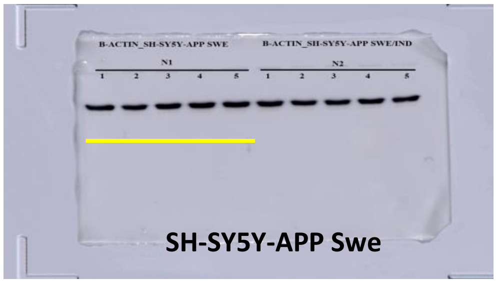

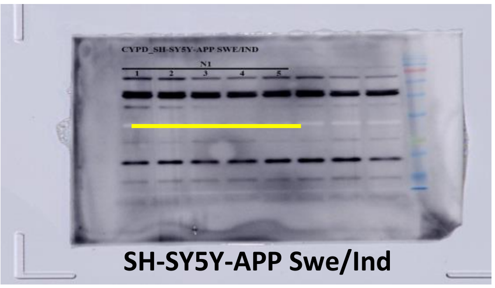

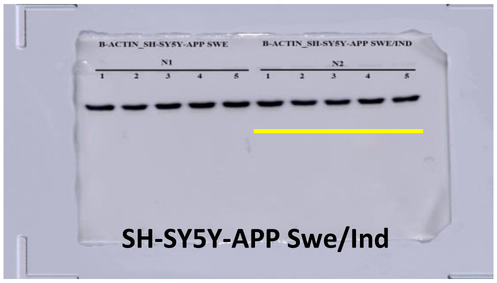

Supplement: Supplementary file 1 — Supplementary Dataset 1. [file 41598_2020_65570_MOESM1_ESM.pdf]
